# Associations of Dietary Intakes of Carotenoids and Vitamin A with Lung Cancer Risk in a Low-Income Population in the Southeastern United States

**DOI:** 10.3390/cancers14205159

**Published:** 2022-10-21

**Authors:** Yan Sun, Jie Wu, Hyung-Suk Yoon, Maciej S. Buchowski, Hui Cai, Stephen A. Deppen, Mark D. Steinwandel, Wei Zheng, Xiao-Ou Shu, William J. Blot, Qiuyin Cai

**Affiliations:** 1 Division of Epidemiology, Department of Medicine, Vanderbilt Epidemiology Center, Vanderbilt-Ingram Cancer Center, Vanderbilt University School of Medicine, 1161 21st Avenue South, Nashville, TN 37232, USA; 2 Division of Gastroenterology, Hepatology and Nutrition, Department of Medicine and Vanderbilt Epidemiology Center, Vanderbilt University School of Medicine, Nashville, TN 37232, USA; 3 Department of Thoracic Surgery, Vanderbilt University Medical Center, Veterans Affairs Hospital, Tennessee Valley VA Healthcare System, Nashville, TN 37212, USA; 4 International Epidemiology Field Station, Vanderbilt Institute for Clinical and Translational Research, Rockville, MD 20850, USA

**Keywords:** lung cancer, African American, carotenoids, vitamin A

## Abstract

**Simple Summary:**

Lung cancer is the second most common cancer and the leading cause of cancer death in the U.S. It is critical to identify the risk factors for lung cancer. However, previous results on the associations of dietary carotenoids and vitamin A intakes with lung cancer risk were inconclusive, and the study populations were mainly European descendants and Asians. This study aimed to prospectively investigate the associations among low-income African Americans and European Americans using resources from the Southern Community Cohort Study. Our findings suggested positive associations between dietary vitamin A intake and lung cancer risk among current smokers and racial-specific associations between dietary vitamin A intake and adenocarcinoma risk. Our study may contribute to understanding the role of nutrient intakes and lung cancer associations among the underrepresented study populations and improving the risk assessment of lung cancer risk.

**Abstract:**

Observational studies found inverse associations of dietary carotenoids and vitamin A intakes with lung cancer risk. However, interventional trials among high-risk individuals showed that β-carotene supplements increased lung cancer risk. Most of the previous studies were conducted among European descendants or Asians. We prospectively examined the associations of lung cancer risk with dietary intakes of carotenoids and vitamin A in the Southern Community Cohort Study, including 65,550 participants with 1204 incident lung cancer cases. Multivariate Cox regression was used to estimate hazard ratios (HRs) and 95% confidence intervals (CIs). Lung cancer cases had lower energy-adjusted dietary intakes of all carotenoids and vitamin A than non-cases. However, dietary intakes of carotenoids and vitamin A were not associated with overall lung cancer risk. A significant positive association of dietary vitamin A intake with lung cancer risk was observed among current smokers (HR_Q4 vs. Q1_ = 1.23; 95% CI: 1.02–1.49; P_trend_ = 0.01). In addition, vitamin A intake was associated with an increased risk of adenocarcinoma among African Americans (HR_Q4 vs. Q1_ = 1.55; 95%CI: 1.08–2.21; P_trend_ = 0.03). Dietary lycopene intake was associated with an increased risk of lung cancer among former smokers (HR_Q4 vs. Q1_ = 1.50; 95% CI: 1.04–2.17; P_trend_ = 0.03). There are positive associations of dietary β-cryptoxanthin intake with squamous carcinoma risk (HR_Q4 vs. Q1_ = 1.49; 95% CI: 1.03–2.15; P_trend_ = 0.03). Further studies are warranted to confirm our findings.

## 1. Introduction

Lung cancer is the leading cause of cancer-related death among men and women in the United States (U.S.) [1]. It is estimated that there have been 236,740 new lung cancer cases and 130,180 lung cancer death in the U.S. in 2022. African Americans (AAs), especially AA males, experienced the highest incidence of lung cancer of any racial or ethnic group in the U.S. [2,3]. There are also substantial disparities in lung cancer risk by socioeconomic status (SES). Socioeconomically disadvantaged populations experience a disproportionate burden of lung cancer compared with more affluent populations. It has been reported that people living in impoverished neighborhoods have a 1.3-fold to 2.2-fold increased risk of lung cancer compared with those living in better neighborhoods [4,5,6,7,8]. However, the biological mechanisms underlying the increased lung cancer risk for AAs and low SES population are unclear.

Carotenoids belong to a class of natural pigments, from yellow and orange to red, synthesized by photosynthetic organisms such as plants, algae, and some bacteria [9]. In the human body, some carotenoids with unmodified β-ionone rings, such as α-carotene, β-carotene, and β-cryptoxanthin, can be metabolized into C20 retinoids and vitamin A (all-trans-vitamin A) in the intestine or liver and are commonly called provitamin A carotenoids [10]. Other carotenoids, such as lycopene, lutein, and zeaxanthin, cannot be metabolized into vitamin A [11]. Carotenoids cannot be produced by humans and must be obtained from diet or supplements. Vegetables and fruits are the principal sources of carotenoids and were found independently inversely associated with lung cancer risk in previous studies [12]. The anti-cancer effects of carotenoids primarily come from their antioxidant properties, which neutralize free radicals and reactive oxygen species [13,14].

Studies have been conducted to evaluate the associations of carotenoids intakes with cancer risks, including lung cancer. A meta-analysis of 19 observational studies suggested that higher levels of β-carotene and vitamin A intake were significantly associated with reduced lung cancer risk [15]. Inverse associations were observed in both case–control and prospective studies. Pooled results from seven cohort studies showed that higher levels of α-carotene, β-carotene, β-cryptoxanthin, lycopene, and lutein/zeaxanthin were associated with a decreased lung cancer risk. However, the association remained significant only for β-cryptoxanthin intake after using the multivariate adjustment model [16]. Unlike observational studies, intervention studies using β-Carotene supplements showed positive associations among the high-risk population and null associations in other trials. The α-Tocopherol, β-Carotene Cancer Prevention Study (ATBC) and Carotene and Vitamin A Efficacy Trial (CARET), the most representative intervention studies of β-carotene supplements, found strong positive associations between β-carotene supplements and lung cancer risk among current smokers and asbestos-exposed workers [17].

It has been shown that intake levels of carotenoids differ between sexes and races [18]. Previous studies were conducted in primarily White and Asian populations. No results have been reported focusing specifically on African Americans (AAs) and low-income populations. We conducted a study within the Southern Community Cohort Study (SCCS), a predominantly low-income AA and European American (EA) population, to investigate the associations between dietary intakes of carotenoids and vitamin A with lung cancer risk.

## 2. Materials and Methods

Study population: The SCCS is a population-based, prospective cohort study of nearly 86,000 English-speaking participants ages 40–79 enrolled from 2002 to 2009 across 12 southeastern states (Alabama, Arkansas, Florida, Georgia, Kentucky, Louisiana, Mississippi, North Carolina, South Carolina, Tennessee, Virginia, and West Virginia) [19]. Approximately 85% of the participants were enrolled from 71 community health centers (CHC), and the remaining 15% were recruited from the general population (2004–2006). The SCCS is a well-characterized cohort: of the participants, 70% were from lower socioeconomic strata, about 60% had less than USD 15,000 annual household income, and about 70% were AAs. The participants completed a computer-assisted, in-person interview or mailed questionnaires at the baseline [19]. The questionnaires included demographic characteristics, tobacco use, family history of cancer, physical activity, dietary intake using the food frequency questionnaire (FFQ), and more [20].

Incident lung cancer cases were ascertained through linkage to state cancer registries using International Classification of Disease Oncology (ICD-O-3) codes C340–C349. By 31 December 2016, 1861 incident lung cancer cases were identified among 84,508 participants. We excluded subjects who (1) reported having ever been diagnosed with cancer of any kind at baseline, including unknown (*n* = 9297); (2) did not have dietary intake information (*n* = 4640); (3) had missing information for the following covariates: race, chronic obstructive pulmonary disease (COPD), smoking status, pack years of smoking, body mass index (BMI), alcohol intake, education, and income levels (*n* = 3497); and (4) were diagnosed with lung cancer (*n* = 203), died, or lost to follow up within the first two years (*n* = 1321). Finally, 65,550 subjects with 1204 lung cancer cases were included in the analysis.

Dietary assessment: To estimate food intake accurately and take sex, race, and regional variations into consideration, the SCCS FFQ was developed from the 24-hour dietary recalls included in the National Health and Nutrition Examination Survey (NHANES) and the Continuing Survey of Food Intakes by Individuals (CSFII) surveys. The dietary intake assessments were derived from the United States Department of Agriculture (USDA) and the University of Minnesota Nutrition Coordinating Center food composition databases. At the baseline, the SCCS used the FFQ to obtain the intake frequency of 104 foods, including 13 food groups, energy, and 18 nutrients [21]. The amount of food items consumed was estimated from frequency and portion size. Participants recalled their food consumption during the past year from the 9 frequency categories: never, rarely, 1/month, 2–3/month, 1/week, 2–3/week, 4–6/week, 1/day, and 2+/day. The FFQ did not elicit portion size details; instead, it used average estimates applied in the NHANES III, NHANES (1999–2004), and CSFII samples [22]. In our study, we evaluated vitamin A and the six most common carotenoids in the Western diet, including α-carotene, β-carotene, β-cryptoxanthin, lutein/zeaxanthin, and lycopene. The daily carotenoid intakes from food sources were estimated separately from dietary supplements.

Statistical analysis: All carotenoids and vitamin A dietary intakes were adjusted for total energy intake and expressed as µg/1000 Kcal. Given the different intake levels between females and males, and AAs and EAs, we used sex- and race-specific quartiles cutoff for dietary intake variables in the analyses. Cox proportional hazards regression analyses were used to calculate the hazard ratios (HRs) and corresponding 95% confidence intervals (CIs) to estimate the associations of dietary intakes of carotenoids and vitamin A with lung cancer risk. We generated three models to evaluate the associations. In the minimum adjusted model (Model 1), we only adjusted for age, race (EA, AA, and others), and sex. In the smoking behavior adjusted model (Model 2), we additionally adjusted for smoking status (current, former, and never smokers), pack years of cigarette smoked (packs/day * years smoked; continuous), enrollment type (CHC or general population), and dietary energy intake. In the fully adjusted model (Model 3), we further adjusted for education (<high school, completed high school, >high school), total household income (<USD 15,000, ≥SD 15,000 and <USD 50,000, ≥USD 50,000), BMI (continuous), chronic obstructive pulmonary disease (COPD) status (ever told by a doctor you have had emphysema or chronic bronchitis: yes/no), and alcohol consumption (number of drinks/days; continuous). We conducted stratification analysis with fully adjusted models by sex (females or males), race (EAs or AAs; other racial groups were excluded due to the small sample size), smoking status (current, former, or never smokers), lung cancer subtypes (adenocarcinoma, squamous cell carcinoma, or small cells lung cancer), and BMI categories. 

## 3. Results

Table 1 presents demographic and major lung cancer risk factors among the study population. About 68% of participants were AAs, and about 60% were women. The mean age was 55.1 years for cases and 51.6 years for non-cases. Compared to non-cases, lung cancer cases were more likely to be males and current smokers, reporting significantly higher pack years. Lung cancer cases had a lower BMI, a lower education level, a lower income level, a higher prevalence of COPD, a higher total energy intake, and consumed more alcohol. Lung cancer cases had lower vegetable and fruit intake and energy-adjusted dietary intakes of all carotenoids (α-carotene, β-carotene, β-cryptoxanthin, lutein and zeaxanthin, and lycopene) and vitamin A (RAE) than non-cases (Table 1).

We first evaluated the associations of dietary carotenoids and vitamin A intakes with lung cancer risk in the minimum adjusted model (Model 1), adjusting for age, race, and sex (Table 2). We observed significant inverse associations between all participants’ dietary intakes of all carotenoids and vitamin A with lung cancer risk. When comparing the highest with the lowest quartiles, the minimum-adjusted HRs_Q4 vs. Q1_ (95% CI) were 0.68 (0.58–0.80; P_trend_ < 0.001) for α-carotene, 0.74 (0.63–0.87; P_trend_ < 0.001) for β-carotene, 0.61 (0.52–0.71; P_trend_ < 0.001) for β-cryptoxanthin, 0.77 (0.65–0.90; P_trend_ < 0.001) for lutein/zeaxanthin, 0.81 (0.70–0.95; P_trend_ = 0.003) for lycopene, and 0.76 (0.64–0.89; P_trend_ < 0.001) for vitamin A. The inverse associations became nonsignificant after additionally adjusting for smoking status, pack years, enrollment type, and energy intake in the smoking behavior models (Model 2). Results were similar in our fully adjusted models, with further adjustment of education, income, COPD status, BMI, and alcohol consumption (Model 3) (Table 2). The results did not change when we added vegetable and fruit intake in model 3. There is a suggestive positive association of dietary vitamin A intake with lung cancer risk with the fully adjusted HR_Q4 vs. Q1_ of 1.14 (95% CI: 0.96–1.35; P_trend_ = 0.08). We also performed analysis among participants recruited from CHC only. The associations were similar to those including all participants.

We then evaluated whether the association of dietary intakes of carotenoids and vitamin A with lung cancer differed by race or sex. No significant associations were detected in the stratification analyses for AAs or EAs or for males or females using the fully adjusted models (Table 3). In addition, no significant associations between dietary intakes of carotenoids and lung cancer risks were observed in the analyses stratified by BMI categories.

We further evaluated whether smoking status modifies these associations. As shown in Table 4, a significant positive association was observed between dietary vitamin A intake and lung cancer risk among current smokers (HR_Q4 vs. Q1_ of 1.23; 95% CI: 1.02–1.49; P_trend_ = 0.01). Additionally, a significant positive association was found between lycopene intake and lung cancer risk among former smokers (HR_Q4 vs. Q1_ of 1.50; 95% CI: 1.04–2.17; P_trend_ = 0.03) (Table 4). We further evaluated whether dietary intakes of carotenoids with lung cancer risk varied by smoking intensity. We did not find any positive associations of β-carotene or other carotenoids with lung cancer risk among heavy smokers.

We conducted analyses stratified by major lung cancer histological types (adenocarcinoma and squamous cell carcinoma). A positive association between dietary β-cryptoxanthin intake and squamous cell carcinoma risk was found with the fully adjusted HR_Q4 vs. Q1_ of 1.49 (95% CI: 1.03–2.15; P_trend_ = 0.03) (Table 4). This positive association was observed in both AAs and EAs, although the associations were not significant due to smaller sample sizes. Vitamin A intake was positively associated with adenocarcinoma risk among AAs (HR_Q4 vs. Q1_ = 1.55; 95%CI: 1.08–2.21; P_trend_ = 0.03), but not among EAs (P_interaction_ = 0.02) (Table 5). In addition, we found dietary intakes of carotenoids were associated with increased adenocarcinoma risks among AAs, but reduced risks among EAs, with significant interactions between EAs and AAs for α-carotene (P_interaction_ = 0.04), β-carotene (P_interaction_ = 0.05), and lycopene (P_interaction_ = 0.05). We did not find significant associations between dietary carotenoids and vitamin A intakes with squamous cell carcinoma risk and the associations did not differ between AAs and EAs (Table 5).

## 4. Discussion

In this large prospective cohort study conducted in low-income AA and EA populations, we found that dietary intakes of carotenoids and vitamin A were not associated with overall lung cancer risk after adjusting for potential confounding factors. Most of the previous observational studies reported inverse associations between specific carotenoids and lung cancer risk. For example, Michaud et al. observed that intake of α-carotene may reduce the risk of lung cancer, especially among women and never smokers, based on pooled results from the Nurses’ Health Study and the Health Professionals Follow-up Study [23]. Marchand et al. found that α-carotene, β-carotene, and lutein were significantly associated with lower lung cancer risk among Hawaiian study participants with a case–control study design [24]. Dorgan et al. showed inverse associations of total carotenoid intake with lung cancer risk among white females and smokers [25]. Similar associations were found by Stefani et al. study that reported inverse dose–response associations between lung cancer risk and α-carotene, β-carotene, lutein, and β-cryptoxanthin but not lycopene intake in Uruguayan men [26]. Our results were different from the conclusions of the Yu et al. meta-analysis [15]. They found significant inverse associations of dietary vitamin A and β-carotene intake with lung cancer risk, especially among Asian study populations and cohort studies. It is difficult to compare their results with ours because of the very different racial component, smoking habits, and food culture. Additionally, this study included about 45% case–control studies in the analysis and showed their concerns about the heterogeneity test results.

Our findings were mainly consistent with the Männistö et al. study; the pooled results of seven cohort studies in North America and Europe [16]. In that study, significant inverse associations were found between intakes of α-carotene, β-carotene, β-cryptoxanthin, lycopene, and lutein/zeaxanthin and lung cancer risk in the age-adjusted model. However, the association remained significant only for β-cryptoxanthin after using the multivariate model. Gallicchio et al., in a systematic review, found similar weak inverse or mostly nonsignificant associations between intakes of carotenoids and lung cancer risk. They also observed similar results in the smoking status stratified analysis [10]. However, we tested the interactions between smoking status and dietary carotenoids and vitamin A intakes and found that the P_interaction_ values were less than 0.05 for all but β-cryptoxanthin. Besides observed significant positive associations for vitamin A (P_interaction_ = 0.03) among current smokers and lycopene (P_interaction_ = 0.04) among former smokers, there were suggestive positive associations for α-carotene (P_interaction_ = 0.01), β-carotene (P_interaction_ = 0.02), and lutein/zeaxanthin (P_interaction_ = 0.01) among current smokers, indicating the different effects among former and never smokers. The observed significant interactions between smoking status and carotenoids and vitamin A intake in our study may result from the distinct smoking behaviors and diverse diet habits of our study population that was characterized by low-income groups and about 70% AAs. 

In a Canadian case–control study, Shareck et al. found that dietary intakes of β-cryptoxanthin and lycopene were inversely associated with the risk of squamous cell carcinoma and small cell carcinoma [27]. In our study, we found positive associations between dietary intake of β-cryptoxanthin and the risk of squamous cell carcinoma. The inconsistent results between the two studies may be due to the differences in study population and dietary intake levels. In the Shareck et al. study, over 80% of cases were Europeans, which is very unlike the racial composition of our participants. Additionally, there were considerable differences in the unadjusted β-cryptoxanthin intake. For example, the median values were 86 μg/day for male cases and 76 μg/day for female cases in the Shareck et al. study. The corresponding values in our study were 151.6 and 128.5 μg/day. Given the small sample size of squamous cell carcinoma cases, our findings need to be verified in a study with a larger sample size.

We found that higher dietary intake of vitamin A was associated with an increased adenocarcinoma risk and the association differed by race; it was significant among AAs but not among EAs. Similar patterns in the associations were observed for α-carotene, β-carotene, and lycopene. The biological mechanisms underlying this association are unknown. Because of relatively small sample sizes in lung cancer histology subgroup analyses, further studies with a large sample size are warranted to confirm our findings.

Previous intervention studies found that β-carotene supplement was associated with incased lung cancer risk among heavy smokers [28,29]. We evaluated whether dietary intakes of carotenoids and lung cancer risk varied by smoking intensity. We defined heavy smokers as current and former smokers who reported using over 20 cigarette pack years but excluded former smokers who had quit smoking for over 15 years [30,31]. We did not find any positive associations of β-carotene or other carotenoids with lung cancer risk among heavy smokers. Several potential explanations exist for the inconsistent results from previous intervention studies and our study. Firstly, the intakes of β-carotene used in the observational studies were much lower than those used in the intervention studies. Based on several national nutrition surveys, the β-carotene dietary intake from the FFQ was less than 3 mg/day [32]. For example, the mean energy-adjusted dietary intake of β-carotene was 1758 µg/day in the ATBC control group and 6382 µg/day in male participants from the New York State Cohort [16]. However, in the intervention studies, the β-carotene supplement was given at 30 mg/day in the Carotene and Retinol Efficacy Trial (CARET). Several hypotheses were proposed to explain the increased lung cancer risk among heavy smokers with high-dose β-carotene supplements. A high concentration of β-carotene may cause excess retinoic signaling, leading to uncontrolled lung epithelial cell differentiation and proliferation. It has been reported that high-dose β-carotene was likely to induce expression of cytochrome P450 family and down-regulate RARβ protein expression, especially in a cigarette smoke environment. Additionally, it is possible that high-dose β-carotene acts as a pro-oxidant in the high oxidative stress condition caused by cigarette smoke [33,34]. Secondly, it is possible that a specific carotenoid may play a protective role together with other micronutrients, different than when it is by itself alone, or there is a difference between synthetic supplements and natural nutrients. Several previous studies showed inverse associations between fruits and vegetables and lung cancer risk [12,35,36]. Chiefly, Holick et al. used the ATBC cohort study population to evaluate the associations between dietary intakes of carotenoids and lung cancer risk and found inverse associations of fruit and vegetable consumption and carotenoids intakes with lung cancer risk. Moreover, they did not find positive associations between dietary β-carotene and lung cancer risk [37]. It has also been reported that the bioaccessibility and bioavailability of carotenoids would change due to different geographical types of vegetables and fruits, the methods of cooking, fat and fiber intake, and food matrix [38].

Our study has several strengths. The population-based prospective cohort study minimizes potential information bias. We also excluded cases identified within the first two years of follow-up to minimize the possibility of reverse causation and/or the potential influence of preclinical conditions on dietary intake. A comprehensive set of prospectively collected exposures associated with lung cancer risk enabled us to adjust for several confounders. Our study includes a large number of lung cancer cases in AAs and low-income participants. Previous studies have not examined the associations of dietary carotenoids and vitamin A intakes in these understudied populations. To reduce the sex and race residual, we used race- sex- specific quartile cutoffs for the dietary carotenoids and vitamin A intakes in the analysis. We further analyzed the results with general quartile cutoff among all study populations and found the main results did not change. Our study also has some limitations. Although we have adjusted smoking status and pack years of cigarette smoking, residual confounding by smoking cannot be completely ruled out. We could not investigate the associations with the use of carotenoids or vitamin A supplements. In the SCCS, 2.9% of participants did not report whether or not they were using a vitamin A supplement and 90.3% of participants reported never using a supplement. Compared to non-cases, cases had a similar frequency of vitamin A supplement use (*p* = 0.65). It is essential to collect supplemental dietary information in future studies because associations of lung cancer risk with carotenoids and vitamin A may differ by intake sources (from food or supplement). In addition, the observed positive associations of dietary vitamin A intake with adenocarcinoma risk, and of dietary β-cryptoxanthin intake with squamous cell carcinoma risk, need to be interpreted with caution due to the small sample size, especially among certain racial groups. These results need to be replicated in other studies.

## 5. Conclusions

In a prospective cohort study with a large proportion of AAs and low-income populations in the Southeastern United States, we found that dietary intakes of carotenoids (α-carotene, β-carotene, β-cryptoxanthin, lycopene, and lutein/zeaxanthin) were not associated with overall lung cancer risk. We observed a significant positive association of vitamin A intake with lung cancer risk among current smokers and a significant positive association of lycopene intake with lung cancer risk among former smokers. We also found that vitamin A intake was positively associated with adenocarcinoma risk among AAs but not among EAs. Further studies are warranted to confirm our findings.

## Figures and Tables

**Table 1:** Characteristics of study population and dietary intakes of carotenoids and vitamin A by lung cancer incident status, SCCS.

Characteristic	Study Populations		*p*-Value *
	Cases	Non-Cases	
	1204	64,346	
Age (years) (mean ± SD)	55.1 ± 8.5	51.6 ± 8.5	<0.001
Sex (%)			<0.001
Female	49.3	59.6	
Male	50.7	40.4	
Race (%)			0.07
European American	31.1	28.2	
African American	65.8	68.0	
others	3.2	3.8	
BMI (kg/m^2^) (%)			<0.001
<25	44.8	24.8	
25–30	29.6	29.7	
≥30	25.7	45.5	
Education level (%)			<0.001
<High school	40.0	28.4	
High school	33.3	33.5	
>High school	26.7	38.1	
Annual household incomes (%)			<0.001
<USD 15,000	67.6	55.3	
USD 15,000–USD 49,999	29.1	35.8	
≥USD 50,000	3.3	8.9	
Smoking status (%)			<0.001
Current smokers	75.8	41.1	
Former smokers	17.7	21.7	
Never smokers	6.5	37.2	
Pack years (the number of packs of cigarettes smoked/day * smoked years) (mean ± SD)	34.1 ± 25.7	22.4 ± 22.0	<0.001
Ever diagnosed with COPD (%)	15.4	8.4	<0.001
Alcohol consumption (the number of drinks per day), (mean ± SD)	1.7 ± 4.3	1.3 ± 3.6	<0.001
Total energy intake, kcal/day, (mean ± SD)	2699.7 ± 1508.3	2582.0 ± 1458.0	0.006
Vegetable intake, (times/day)			<0.001
<1	12.0	11.0	
1	43.2	37.4	
2	30.6	34.5	
≥3	14.2	17.1	
Fruit or fruit juice intake, (times/day)			<0.001
<1	29.8	23.2	
1	32.9	34.9	
2	19.7	22.9	
≥3	17.7	19.0	
**Dietary carotenoids intake, median (Q1, Q3) (µg/1000 Kcal)**			
α-carotene	134.0 (75.9, 249.7)	150.2 (81.4, 281.5)	<0.001
β-carotene	1370.8 (859.7, 2355.9)	1504.3 (924.5, 2414.4)	0.009
β-cryptoxanthin	56.2 (31.0, 118.3)	67.9 (35.4, 130.9)	<0.001
Lutein and zeaxanthin	1069.9 (670.2, 1869.1)	1137.3 (694.8, 1957.1)	0.04
Lycopene	1856.4 (1280.3, 2791.8)	2012.0 (1353.8, 2866.7)	0.002
Vitamin A, RAE ^&^	353.0 (260.0, 456.7)	353.8 (270.1, 458.7)	0.31

* *p*-values calculation: age, pack years, alcohol consumption, and total energy intake were calculated by *t*-test; sex, race, BMI categories, educational level, annual household incomes, smoking status, and ever diagnosed with COPD, vegetables and fruits/fruit juice intake times per day were calculated by chi-square test; carotenoids and vitamin A intake levels were compared by Wilcoxon rank-sum test. ^&^ RAE is retinol activity equivalents.

**Table 2:** Associations of dietary intakes of carotenoids and vitamin A with lung cancer risk, SCCS.

Carotenoids	Quartiles	Case	Model 1 ^#^	Model 2 ^#^	Model 3 ^#^
			HR (95% CI)	HR (95% CI)	HR (95% CI)
α-carotene	1	334	ref	ref	ref
	2	314	0.87 (0.75–1.01)	0.98 (0.84–1.14)	1.02 (0.87–1.19)
	3	281	0.73 (0.63–0.86)	0.90 (0.76–1.05)	0.96 (0.81–1.12)
	4	275	0.68 (0.58–0.80)	0.95 (0.81–1.12)	1.03 (0.87–1.22)
P_trend_			<0.001	0.33	0.94
β-carotene	1	317	ref	ref	ref
	2	309	0.86 (0.74–1.01)	0.99 (0.84–1.15)	1.02 (0.87–1.19)
	3	275	0.72 (0.61–0.84)	0.89 (0.76–1.05)	0.94 (0.79–1.11)
	4	303	0.74 (0.63–0.87)	1.03 (0.88–1.22)	1.09 (0.92–1.28)
P_trend_			<0.001	0.99	0.55
β-Cryptoxanthin	1	351	ref	ref	ref
	2	314	0.82 (0.71–0.96)	0.98 (0.84–1.14)	1.01 (0.87–1.18)
	3	265	0.66 (0.56–0.77)	0.85 (0.72–1.00)	0.89 (0.76–1.05)
	4	274	0.61 (0.52–0.71)	0.90 (0.76–1.06)	0.94 (0.80–1.11)
P_trend_			<0.001	0.07	0.23
Lutein and	1	321	ref	ref	ref
Zeaxanthin	2	296	0.81 (0.69–0.95)	0.95 (0.81–1.12)	0.99 (0.84–1.16)
	3	277	0.71 (0.60–0.83)	0.89 (0.76–1.05)	0.95 (0.80–1.12)
	4	310	0.77 (0.65–0.90)	0.99 (0.84–1.16)	1.05 (0.89–1.24)
P_trend_			<0.001	0.74	0.66
Lycopene	1	343	ref	ref	ref
	2	317	0.94 (0.80–1.09)	1.01 (0.87–1.18)	1.04 (0.89–1.21)
	3	270	0.81 (0.69–0.95)	0.93 (0.79–1.09)	0.96 (0.82–1.13)
	4	274	0.81 (0.70–0.95)	0.98 (0.83–1.14)	1.01 (0.85–1.18)
P_trend_			0.003	0.53	0.82
Vitamin A, RAE	1	311	ref	ref	ref
	2	285	0.81 (0.69–0.95)	0.93 (0.79–1.09)	0.95 (0.80–1.12)
	3	290	0.76 (0.64–0.89)	0.98 (0.83–1.15)	1.02 (0.87–1.21)
	4	318	0.76 (0.64–0.89)	1.09 (0.93–1.28)	1.14 (0.96–1.35)
P_trend_			<0.001	0.24	0.08

**^#^** Model 1—minimum adjustment model: adjusting for age, race, and sex. Model2—smoking behavior adjustment model: adjusting for age, race, sex, smoking status, packyears, enrollment type, and energy intake. Model 3—fully adjusted model: adjusting for age, race, sex, smoking status, packyears, enrollment type, energy intake, COPD status, education level, income level, alcohol consumption, and BMI.

**Table 3:** Associations of dietary intakes of carotenoids and vitamin A with lung cancer risk by race and sex, SCCS.

		α-Carotene		β-Carotene		β-Cryptoxanthin		Lutein and Zeaxanthin		Lycopene		Vitamin A	
Quartile		Case	HR * (95% CI)	Case	HR (95% CI)	Case	HR (95% CI)	Case	HR (95% CI)	Case	HR (95% CI)	Case	HR (95% CI)
Sex													
Women	1	162	ref	156	ref	188	ref	158	ref	170	ref	149	ref
	2	160	1.07 (0.86–1.34)	147	1.02 (0.81–1.28)	145	0.88 (0.71–1.10)	154	1.07 (0.85–1.34)	144	0.96 (0.77–1.20)	140	0.99 (0.78–1.25)
	3	128	0.87 (0.69–1.10)	141	1.02 (0.81–1.29)	127	0.83 (0.66–1.04)	128	0.92 (0.72–1.16)	142	1.03 (0.82–1.29)	151	1.15 (0.91–1.45)
	4	144	1.11 (0.88–1.40)	150	1.14 (0.90–1.44)	134	0.89 (0.71–1.12)	154	1.07 (0.85–1.35)	138	1.02 (0.82–1.29)	154	1.22 (0.96–1.54)
	P_trend_		0.80		0.29		0.24		0.85		0.72		0.06
Men	1	172	ref	161	ref	163	ref	163	ref	173	ref	162	ref
	2	154	0.97 (0.78–1.21)	162	1.02 (0.82–1.27)	169	1.17 (0.94–1.45)	142	0.92 (0.73–1.15)	173	1.12 (0.91–1.39)	145	0.92 (0.72–1.16)
	3	153	1.05 (0.84–1.32)	134	0.87 (0.68–1.10)	138	0.98 (0.78–1.24)	149	0.98 (0.78–1.23)	128	0.91 (0.72–1.15)	139	0.92 (0.72–1.17)
	4	131	0.95 (0.75–1.21)	153	1.03 (0.81–1.30)	140	1.01 (0.79–1.27)	156	1.03 (0.82–1.30)	136	0.99 (0.78–1.25)	164	1.06 (0.83–1.35)
	P_trend_		0.89		0.84		0.69		0.69		0.52		0.58
Race													
EA	1	123	ref	100	ref	113	ref	102	ref	115	ref	98	ref
	2	92	0.84 (0.64–1.10)	105	1.15 (0.87–1.51)	101	1.08 (0.83–1.42)	96	1.09 (0.82–1.45)	93	0.94 (0.71–1.24)	94	1.05 (0.78–1.40)
	3	80	0.79 (0.60–1.06)	79	0.95 (0.70–1.29)	78	0.88 (0.66–1.18)	84	0.98 (0.73–1.32)	87	1.03 (0.78–1.38)	89	1.04 (0.77–1.40)
	4	79	0.84 (0.63–1.13)	90	1.06 (0.79–1.43)	82	0.93 (0.69–1.24)	92	1.03 (0.77–1.38)	79	0.95 (0.71–1.27)	93	1.05 (0.78–1.43)
	P_trend_		0.19		0.98		0.38		1.00		0.88		0.77
AA	1	203	ref	206	ref	226	ref	209	ref	216	ref	206	ref
	2	211	1.09 (0.90–1.33)	202	0.99 (0.82–1.21)	203	0.98 (0.81–1.19)	194	0.96 (0.79–1.17)	216	1.11 (0.92–1.35)	189	0.93 (0.76–1.15)
	3	193	1.04 (0.85–1.27)	183	0.90 (0.74–1.11)	178	0.90 (0.73–1.10)	184	0.92 (0.75–1.13)	178	0.97 (0.79–1.19)	188	0.98 (0.78–1.21)
	4	185	1.10 (0.90–1.36)	201	1.07 (0.87–1.31)	185	0.95 (0.78–1.17)	205	1.02 (0.84–1.25)	182	1.01 (0.82–1.24)	209	1.12 (0.91–1.38)
	P_trend_		0.48		0.74		0.47		0.91		0.74		0.24

* HR were calculated by fully adjusted model: adjusting for age, race, sex, smoking status, pack years, enrollment type, energy intake, COPD status, education level, income level, alcohol consumption, and BMI.

**Table 4:** Associations of dietary intakes of carotenoids and vitamin A with lung cancer risk by smoking status and lung cancer histological subtypes, SCCS.

		α-Carotene		β-Carotene		β-Cryptoxanthin		Lutein and Zeaxanthin		Lycopene		Vitamin A	
Quartile		Case	HR (95% CI)	Case	HR (95% CI)	Case	HR (95% CI)	Case	HR (95% CI)	Case	HR (95% CI)	Case	HR (95% CI)
Smoking status													
Current smokers	1	269	ref	264	ref	291	ref	257	ref	277	ref	261	ref
	2	237	1.02 (0.86–1.22)	233	0.98 (0.82–1.17)	242	1.01 (0.85–1.20)	225	1.03 (0.86–1.23)	252	1.04 (0.88–1.24)	216	0.94 (0.78–1.13)
	3	203	0.96 (0.80–1.16)	198	0.94 (0.78–1.14)	200	0.92 (0.77–1.11)	204	1.01 (0.83–1.22)	205	0.95 (0.79–1.14)	217	1.09 (0.90–1.31)
	4	204	1.17 (0.97–1.41)	218	1.18 (0.98–1.42)	180	0.93 (0.77–1.13)	227	1.16 (0.97–1.40)	179	0.88 (0.73–1.07)	219	1.23 (1.02–1.49)
	P_trend_		0.22		0.16		0.34		0.15		0.14		0.01
Former smokers	1	48	ref	36	ref	44	ref	45	ref	49	ref	34	ref
	2	53	0.90 (0.61–1.34)	52	1.12 (0.73–1.72)	50	0.99 (0.66–1.49)	47	0.72 (0.48–1.10)	45	1.02 (0.68–1.54)	50	0.95 (0.60–1.49)
	3	55	0.90 (0.60–1.33)	57	0.98 (0.64–1.51)	43	0.72 (0.47–1.10)	54	0.73 (0.49–1.09)	46	1.03 (0.68–1.55)	53	0.83 (0.53–1.30)
	4	57	0.78 (0.52–1.15)	68	0.99 (0.65–1.50)	76	1.06 (0.72–1.55)	67	0.79 (0.53–1.17)	73	1.50 (1.04–2.17)	76	0.91 (0.59–1.39)
	P_trend_		0.22		0.73		0.96		0.39		0.03		0.61
Never Smokers	1	17	ref	17	ref	16	ref	19	ref	17	ref	16	ref
	2	24	1.19 (0.64–2.23)	24	1.08 (0.58–2.03)	22	1.11 (0.58–2.12)	24	1.03 (0.56–1.90)	20	1.15 (0.60–2.19)	19	0.90 (0.45–1.77)
	3	23	1.01 (0.53–1.91)	20	0.77 (0.40–1.50)	22	0.93 (0.48–1.78)	19	0.73 (0.38–1.39)	19	1.07 (0.55–2.07)	20	0.77 (0.39–1.53)
	4	14	0.58 (0.28–1.20)	17	0.58 (0.29–1.17)	18	0.62 (0.31–1.24)	16	0.58 (0.29–1.15)	22	1.17 (0.62–2.23)	23	0.77 (0.39–1.52)
	P_trend_		0.10		0.06		0.11		0.06		0.70		0.42
Histological subtypes													
Adeno- Carcinoma	1	96	ref	99	ref	122	ref	110	ref	110	ref	93	ref
	2	118	1.30 (0.99–1.71)	110	1.14 (0.86–1.50)	100	0.90 (0.69–1.17)	99	0.93 (0.71–1.23)	109	1.07 (0.82–1.39)	104	1.12 (0.84–1.50)
	3	101	1.17 (0.88–1.55)	92	0.97 (0.73–1.30)	82	0.76 (0.57–1.01)	90	0.87 (0.65–1.16)	92	0.95 (0.72–1.26)	99	1.14 (0.85–1.53)
	4	88	1.10 (0.82–1.49)	102	1.14 (0.86–1.53)	99	0.94 (0.71–1.24)	104	1.01 (0.77–1.33)	92	0.97 (0.74–1.29)	107	1.27 (0.94–1.71)
	P_trend_		0.70		0.61		0.41		0.95		0.67		0.13
Squamous cell carcinoma	1	72	ref	69	ref	56	ref	62	ref	66	ref	61	ref
	2	65	0.97 (0.69–1.36)	57	0.86 (0.60–1.23)	62	1.28 (0.89–1.85)	63	1.10 (0.77–1.57)	65	1.15 (0.82–1.63)	54	0.90 (0.61–1.31)
	3	55	0.85 (0.59–1.21)	53	0.81 (0.56–1.18)	65	1.43 (0.99–2.06)	56	0.99 (0.68–1.44)	59	1.18 (0.82–1.68)	62	1.10 (0.76–1.60)
	4	58	1.02 (0.71–1.45)	71	1.14 (0.81–1.62)	67	1.49 (1.03–2.15)	69	1.19 (0.83–1.70)	60	1.22 (0.85–1.75)	73	1.29 (0.89–1.86)
	P_trend_		0.85		0.51		0.03		0.46		0.27		0.10
Small cells	1	50	ref	43	ref	48	ref	38	ref	46	ref	41	ref
	2	41	0.88 (0.58–1.33)	42	1.04 (0.68–1.60)	44	1.06 (0.70–1.60)	32	0.97 (0.60–1.56)	43	1.05 (0.69–1.59)	36	0.91 (0.57–1.43)
	3	40	0.90 (0.59–1.37)	34	0.88 (0.56–1.39)	34	0.88 (0.56–1.37)	47	1.47 (0.95–2.27)	32	0.86 (0.54–1.36)	44	1.18 (0.76–1.84)
	4	24	0.64 (0.39–1.06)	36	1.04 (0.66–1.64)	29	0.81 (0.51–1.30)	38	1.19 (0.75–1.88)	34	0.93 (0.59–1.46)	34	1.00 (0.62–1.61)
	P_trend_		0.12		0.92		0.30		0.21		0.57		0.71

**Table 5:** The associations of dietary carotenoids and vitamin A intakes with risk of adenocarcinoma and squamous cell carcinoma lung cancer by race, SCCS.

Carotenoids	Quartiles	Adenocarcinoma					Squamous Cell Carcinoma				
		EAs		AAs			EAs		AAs		
		Case	HR 95%CI	Case	HR 95%CI	P_interaction_	Case	HR 95%CI	Case	HR 95%CI	P_interaction_
α-carotene	1	37	ref	55	ref	0.04	23	ref	47	ref	0.96
	2	24	0.71 (0.42–1.20)	91	1.71 (1.22–2.40)		27	1.32 (0.75–2.31)	34	0.75 (0.48–1.17)	
	3	24	0.75 (0.44–1.26)	74	1.45 (1.02–2.07)		14	0.76 (0.39–1.48)	39	0.85 (0.55–1.32)	
	4	19	0.64 (0.36–1.13)	65	1.39 (0.96–2.01)		21	1.22 (0.66–2.25)	37	0.95 (0.60–1.48)	
P_trend_			0.13		0.21			0.94		0.91	
β-carotene	1	33	ref	61	ref	0.05	21	ref	44	ref	0.48
	2	27	0.87 (0.52–1.46)	82	1.35 (0.96–1.88)		21	1.12 (0.61–2.08)	36	0.81 (0.52–1.28)	
	3	25	0.85 (0.50–1.44)	64	1.06 (0.74–1.52)		18	1.08 (0.57–2.06)	31	0.66 (0.41–1.06)	
	4	19	0.64 (0.35–1.14)	78	1.39 (0.98–1.98)		25	1.46 (0.80–2.69)	46	1.07 (0.69–1.67)	
P_trend_			0.14		0.20			0.25		0.91	
β-Cryptoxanthin	1	38	ref	80	ref	0.16	16	ref	37	ref	0.76
	2	23	0.70 (0.42–1.18)	73	0.98 (0.71–1.35)		28	2.21 (1.19–4.11)	32	0.94 (0.58–1.52)	
	3	23	0.71 (0.42–1.20)	55	0.76 (0.54–1.08)		20	1.73 (0.88–3.38)	43	1.35 (0.86–2.13)	
	4	20	0.61 (0.35–1.07)	77	1.10 (0.79–1.52)		21	1.76 (0.90–3.44)	45	1.44 (0.91–2.27)	
P_trend_			0.08		0.92			0.19		0.05	
Lutein and	1	37	ref	68	ref	0.09	19	ref	41	ref	0.80
Zeaxanthin	2	25	0.74 (0.44–1.24)	73	1.08 (0.78–1.52)		25	1.55 (0.85–2.83)	34	0.86 (0.54–1.37)	
	3	18	0.54 (0.31–0.96)	70	1.06 (0.75–1.50)		18	1.16 (0.60–2.24)	36	0.89 (0.56–1.42)	
	4	24	0.71 (0.42–1.20)	74	1.13 (0.81–1.60)		23	1.41 (0.75–2.64)	46	1.09 (0.70–1.71)	
P_trend_			0.11		0.52			0.47		0.64	
Lycopene	1	33	ref	72	ref	0.05	24	ref	39	ref	0.55
	2	33	1.11 (0.68–1.81)	72	1.07 (0.77–1.49)		19	0.96 (0.52–1.76)	44	1.34 (0.86–2.08)	
	3	23	0.87 (0.50–1.49)	66	1.02 (0.72–1.42)		21	1.32 (0.72–2.41)	38	1.25 (0.79–1.97)	
	4	15	0.57 (0.31–1.07)	75	1.16 (0.84–1.62)		21	1.29 (0.71–2.36)	36	1.20 (0.75–1.91)	
P_trend_			0.07		0.45			0.28		0.53	
Vitamin A, RAE	1	30	ref	60	ref	0.02	20	ref	38	ref	0.94
	2	28	0.98 (0.58–1.67)	74	1.23 (0.87–1.75)		19	1.04 (0.54–1.99)	35	0.90 (0.56–1.46)	
	3	28	1.03 (0.60–1.77)	67	1.19 (0.82–1.71)		20	1.14 (0.60–2.19)	39	1.09 (0.68–1.77)	
	4	18	0.65 (0.35–1.21)	84	1.55 (1.08–2.21)		26	1.41 (0.75–2.65)	45	1.25 (0.77–2.02)	
P_trend_			0.24		0.03			0.26		0.24	

## Data Availability

Data used in the present study can be requested through the Southern Community Cohort Study online request system (https://ors.southerncommunitystudy.org/, accessed on 1 September 2022).
